# Ebselen alleviates testicular pathology in mice with Zika virus infection and prevents its sexual transmission

**DOI:** 10.1371/journal.ppat.1006854

**Published:** 2018-02-15

**Authors:** Yogy Simanjuntak, Jian-Jong Liang, Si-Yu Chen, Jin-Kun Li, Yi-Ling Lee, Han-Chung Wu, Yi-Ling Lin

**Affiliations:** 1 Institute of Biomedical Sciences, Academia Sinica, Taipei, Taiwan; 2 Institute of Cellular and Organismic Biology, Academia Sinica, Taipei, Taiwan; 3 Genomic Research Center, Academia Sinica, Taipei, Taiwan; NIH, UNITED STATES

## Abstract

Despite the low case fatality, Zika virus (ZIKV) infection has been associated with microcephaly in infants and Guillain-Barré syndrome. Antiviral and vaccine developments against ZIKV are still ongoing; therefore, in the meantime, preventing the disease transmission is critical. Primarily transmitted by Aedes species mosquitoes, ZIKV also can be sexually transmitted. We used AG129 mice lacking interferon-α/β and -γ receptors to study the testicular pathogenesis and sexual transmission of ZIKV. Infection of ZIKV progressively damaged mouse testes, increased testicular oxidative stress as indicated by the levels of reactive oxygen species, nitric oxide, glutathione peroxidase 4, spermatogenesis-associated-18 homolog in sperm and pro-inflammatory cytokines including IL-1β, IL-6, and G-CSF. We then evaluated the potential role of the antioxidant ebselen (EBS) in alleviating the testicular pathology with ZIKV infection. EBS treatment significantly reduced ZIKV-induced testicular oxidative stress, leucocyte infiltration and production of pro-inflammatory response. Furthermore, it improved testicular pathology and prevented the sexual transmission of ZIKV in a male-to-female mouse sperm transfer model. EBS is currently in clinical trials for various diseases. ZIKV infection could be on the list for potential use of EBS, for alleviating the testicular pathogenesis with ZIKV infection and preventing its sexual transmission.

## Author summary

Male-to-female sexual transmission of ZIKV has been reported more than the other sexual modes. Here we observe the disease progression of ZIKV in the testicular tissue of mouse models by gaining access to interstitial cells, the basement membrane of seminiferous tubule, spermatogenic cells, and sperm. In addition, we develop an animal model to study male-to-female sexual transmission with a high transmissibility through seminal transfer that allows us to identify a possible therapeutic intervention. Our data indicate that testicular oxidative stress and pro-inflammatory response may contribute to testicular pathology as well as successful sexual transmission of ZIKV; thus, this could serve as a potential target for therapeutic purpose. We evaluated several potential antioxidants and found that ebselen (EBS) treatments in male mice display a better property to alleviate testicular pathogenesis and prevent seminal transmission of ZIKV than vitamin C or quercetin.

## Introduction

Zika virus (ZIKV) is a single-stranded RNA virus that belongs to the *Flaviviridae* family [1]. The recent outbreak of ZIKV infection has created a public health emergency of international concern [2]. ZIKV infection displays nonspecific clinical features and generally causes mild symptoms in humans [1]. Although the case fatality with ZIKV infection is low, its infection has been linked to congenital microcephaly and Guillain-Barré syndrome [2, 3]. Currently, there is no approved antiviral drug or vaccines for ZIKV infection. Preventing ZIKV transmission is a significant strategy for disease control and management.

ZIKV is transmitted to people primarily via the bite of an infected Aedes species mosquito [1]. However, recent studies have suggested the sexual transmission of ZIKV in humans [4–6]. Infectious ZIKV in semen has been reported [7, 8]. Moreover, unlike in serum or urine samples, ZIKV RNA can still be detected in semen up to 62 to 188 days after the onset of symptoms [9–11]. Studies have reported more male to female transmission than with other sexual modes [12]. Notably, sexual transmission of ZIKV may occur before, during, or after the onset of symptoms [2]. This may suggest the complexity of risk factors for sexually transmitted ZIKV. Other indications that ZIKV is sexually transmissible come from animal studies. ZIKV infects and damages the testes of infected mice, which results in the loss of germ cells, degeneration of seminiferous epithelium, and poor-quality sperm [13, 14].

Oxidative stress plays an important role in the pathogenesis of both RNA and DNA viruses [15, 16]. Notably, in many cases of testicular dysfunction and infertility, oxidative stress appears to be a common underlying factor [17, 18]. Ebselen (EBS), an antioxidant currently in clinical trials for preventing and treating various disorders, has been shown to reduce oxidative stress and improve histopathological features in a testicular injury study [19, 20]. EBS catalyzes the reduction of reactive oxygen species (ROS) in a mode similar to glutathione peroxidase 4 (GPx4) [21]. GPx4 is highly expressed in spermatogenic cells and plays a dual role as an antioxidant enzyme and a structural protein [22]. In addition, EBS plays a role in inhibiting the catalytic activity of nitric oxide synthase (NOS); thus, it may also reduce the level of nitric oxide (NO) and NO-associated inflammatory cytokines [23, 24].

Here, we demonstrate that ZIKV increased testicular oxidative stress and pro-inflammatory response. Moreover, we propose a possible therapeutic intervention by using the antioxidant EBS to alleviate the testicular pathogenesis and prevent the sexual transmission of ZIKV.

## Results

### ZIKV infects and damages testes

**Fig 1:**
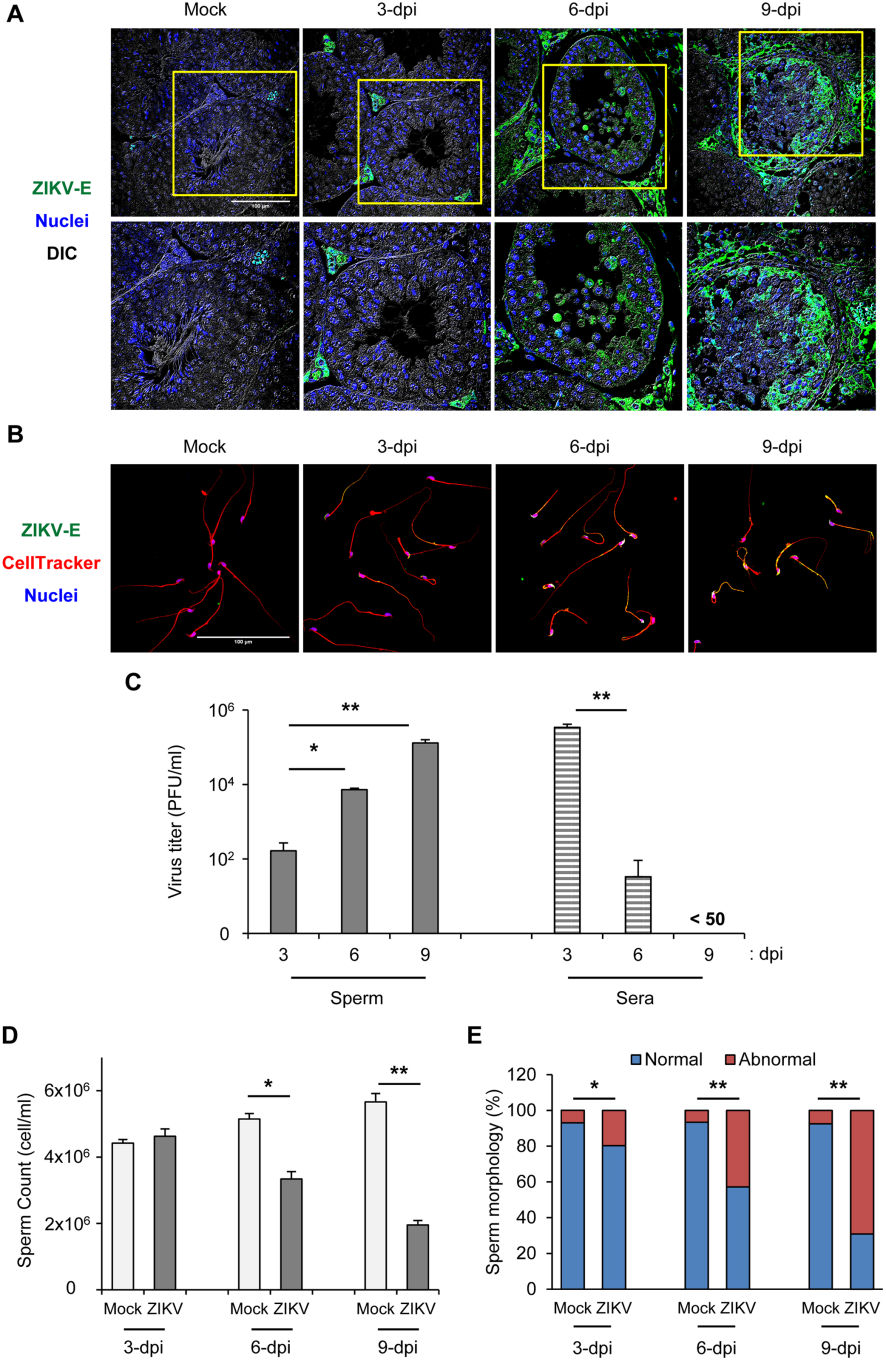
ZIKV infects and damages testes. AG129 mice were subcutaneously infected in the footpad with 5x10^4^ plaque-forming units (PFU)/mouse of ZIKV. Testes, sperm, and sera were collected on 3, 6, and 9 days post-infection (dpi). (A) Confocal microscopy of testes sections immunostained for ZIKV-E (green) and Hoechst for nuclei (blue). (B) Confocal microscopy of sperm immunostained for ZIKV-E (green), CellTracker for cytoplasm (red), and Hoechst for nuclei (blue). Differential interference contrast (DIC). Scale bar: 100 μm. (C) Plaque-forming assay of viral load in sperm and sera. (D) Total number of sperm (cells/ml). (E) Proportion of sperm with normal and abnormal morphology. Data are mean ± SD (n = 6 mice/group). *P<0.05 and **P<0.01 by Mann-Whitney U test.

AG129 mice lacking interferon-α/β and -γ receptors were challenged with ZIKV (strain PRVABC59) by subcutaneous route in the footpad and the progression of ZIKV infection in the testes and sperm was prospectively evaluated. On 3 days post-infection (dpi), ZIKV envelope protein (ZIKV-E) was found predominantly in interstitial cells and less so in the basement membrane of the seminiferous tubule (SNT) (Fig 1A; 3-dpi). On 6 dpi, ZIKV-E was detected in interstitial cells, the basement membrane of the SNT, and in spermatogenic cells (Fig 1A; 6-dpi). Notably, ZIKV-E expression in spermatogenic cells was even more prominent on 9 dpi (Fig 1A; 9-dpi). Autofluorescence of red blood cells was noted in histological sections, including in mock-infected testes, as previously reported, although we used paraformaldehyde fixation to minimize the interference [25]. It is intriguing to speculate that testicular infection of ZIKV is initially presented in interstitial cells adjacent to the endothelium and then spread to the basement membrane of the SNT to further infect the spermatogenic cells. In addition, ZIKV-E and double-stranded RNA (dsRNA) were detected in the head, middle piece, and tail of sperm of infected mice as early as 3 dpi (Fig 1B and S1 Fig). Moreover, infectious ZIKV was detected in sperm using a plaque-forming assay. The level of infectious ZIKV in the sperm gradually increased from approximately 2 log_10_ plaque-forming units (PFU)/ml on 3 dpi to 5 log_10_ PFU/ml on 9 dpi (Fig 1C, gray bar). In agreement with a previous study [13], the viremia level was significantly decreased and undetectable on 6 and 9 dpi, respectively (Fig 1C, striped bar). These data indicate that ZIKV infects testes and sperm of mice.

We also evaluated the pathological features of ZIKV infection in testes. ZIKV infection on 3 dpi did not greatly affect the histological structure of testis as compared with age-matched mock controls. The architecture of the SNT remained intact, with normal germinal epithelium and accumulation of sperm in the lumen (S2A Fig). On 6 and 9 dpi, ZIKV infection caused pathological features in the testis, including involution of the SNT, degeneration of spermatocytes, and the appearance of multinucleated giant cells in the lumen (S2A Fig).

In addition, we checked the expression of TRA98 and ETV5. TRA98 is a germ cell marker, whereas ETV5 plays a role in mediating the blood—testis barrier (BTB) [26, 27]. In agreement with previous study [13], the expression of TRA98 and ETV5 was gradually impaired by ZIKV infection (S2B Fig). TRA98 and ETV5 expression was reduced on 6 and 9 dpi as compared with controls (S2B Fig). These testicular pathological features suggest that ZIKV may impair spermatogenesis.

To confirm this notion, we evaluated sperm quality in terms of sperm count and morphology [28]. ZIKV did not affect sperm count on 3 dpi, but on 6 and 9 dpi, sperm number was significantly lower in ZIKV-infected than control mice (Fig 1D). Also, as early as 3 dpi, the proportion of abnormal sperm was greater in ZIKV-infected than controls (Fig 1E) and was even greater on 6 and 9 dpi in ZIKV-infected mice (Fig 1E). Confocal images showed abnormal sperm with short tail, hairpin tail, and bent middle piece in ZIKV-infected mice (S2C Fig). Overall, these data suggest that ZIKV infection damages testes and reduces sperm quality.

### ZIKV infection increases testicular oxidative stress and inflammatory response

**Fig 2:**
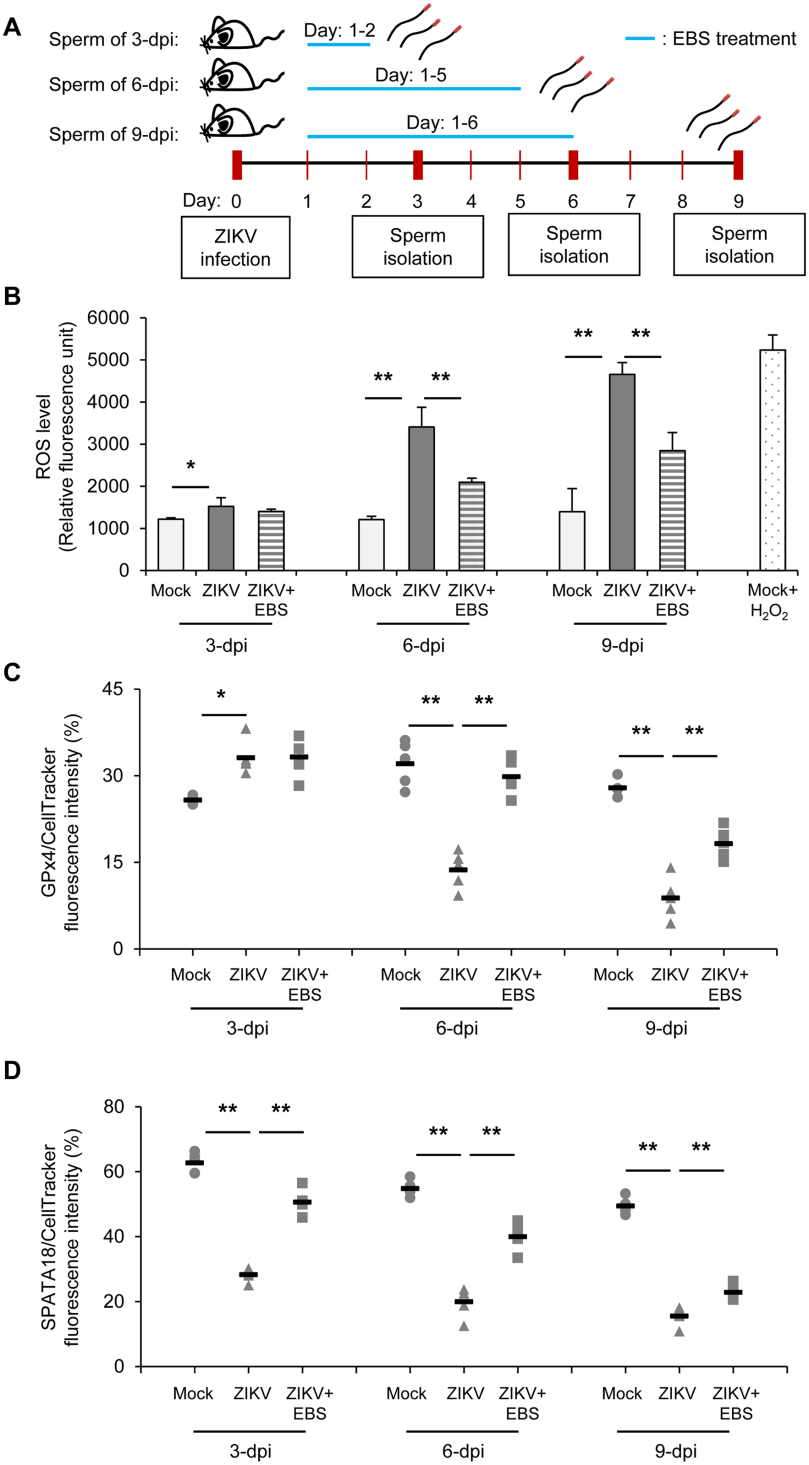
ZIKV infection increases testicular oxidative stress. (A) Schematic experimental design. Mice were subcutaneously infected in the footpad with 5x10^4^ PFU/mouse of ZIKV. Mice were intraperitoneally treated with the antioxidant ebselen (EBS; 10 mg/kg body weight/mouse/day) or solvent control on day 1–2 (group: 3-dpi), day 1–5 (group: 6-dpi), or day 1–6 (group: 9-dpi) after infection. Sperm was collected on 3, 6, and 9 dpi. (B) Intracellular reactive oxygen species (ROS) assay. ROS levels in sperms were measured by use of the OxiSelect intracellular ROS indicator, with H2O2-treated sperms as a positive control. Relative fluorescence intensity was determined by use of a fluorescence plate reader. (C) Relative fluorescence intensity of GPx4 to CellTracker. (D) Relative fluorescence intensity of SPATA18 to CellTracker. Data are mean ± SD (n = 6 mice or 5 random confocal fields/group). *P<0.05 and **P<0.01 by Kruskal-Wallis, Bonferroni post-hoc test.

Taking into account that reactive oxygen species (ROS) play a role in viral pathogenesis as well as testicular pathology [15–18], we measured whether ZIKV infection increases testicular oxidative stress. Semen parameters have been widely used to gauge testicular pathology associated with oxidative stress [18]. A high level of ROS in semen is linked to poor sperm quality [29]. Moreover, a low expression of antioxidant enzymes including GPx4 is associated with abnormal spermatogenesis [30, 31]. We prospectively evaluated testicular oxidative stress of ZIKV-infected mice with or without treatment with the antioxidant ebselen (EBS) as outlined in Fig 2A. EBS at 10 mg/kg body weight (bw) has been shown to reduce testicular oxidative stress and testicular damage in animal study [20]. ROS levels were higher in sperm from ZIKV-infected mice than controls as early as 3 dpi (Fig 2B; gray vs. white bar). Notably, therapeutic treatments with EBS (10 mg/kg bw/mouse/ip/day) after ZIKV challenge significantly decelerated the elevated ROS levels in sperm on 6 and 9 dpi (Fig 2B, striped vs. gray bar). We further evaluated the level of nitric oxide (NO) in sperm because EBS has been demonstrated to inhibit the catalytic activity of nitric oxide synthase [23]. Consistently, ZIKV infection significantly increased NO level in sperm and treatment with EBS could repress the level (S3A Fig).

Moreover, the expression of the scavenging enzyme GPx4, the most highly expressed GPx isoform that supports the middle piece structure of sperm [22], was also affected by ZIKV infection. Control sperm showed normal morphology with expression of GPx4 in the middle piece (S3B Fig). ZIKV infection increased GPx4 expression in sperm on 3 dpi, presumably due to the altered cellular response associated with viral replication (Fig 2C and S3B Fig; Mock vs. ZIKV). However, on 6 and 9 dpi, GPx4 expression was lower in infected than control sperm (Fig 2C and S3B Fig). Remarkably, sperm of ZIKV-infected mice receiving EBS treatment showed significantly improved GPx4 expression on 6 and 9 dpi (Fig 2C and S3B Fig; ZIKV vs. ZIKV+EBS). In addition, we evaluated the spermatogenesis-associated-18 homolog (SPATA18/MIEAP) that also mediates both oxidative stress and structural stability of sperm. SPATA18 is involved in eliminating oxidized mitochondrial proteins and reducing ROS generation [32, 33]. In addition, poor expression of SPATA18 adversely affects the spermatogenesis and morphology of sperm [34, 35]. SPATA18 was detected in the head, middle piece and upper tail of control sperm but its expression was lower in sperm of ZIKV-infected mice as early as 3 dpi (Fig 2D and S3C Fig; Mock vs. ZIKV). This adverse effect of ZIKV infection on SPATA18 expression could be significantly alleviated by EBS treatment (Fig 2D and S3C Fig; ZIKV vs. ZIKV+EBS).

**Fig 3:**
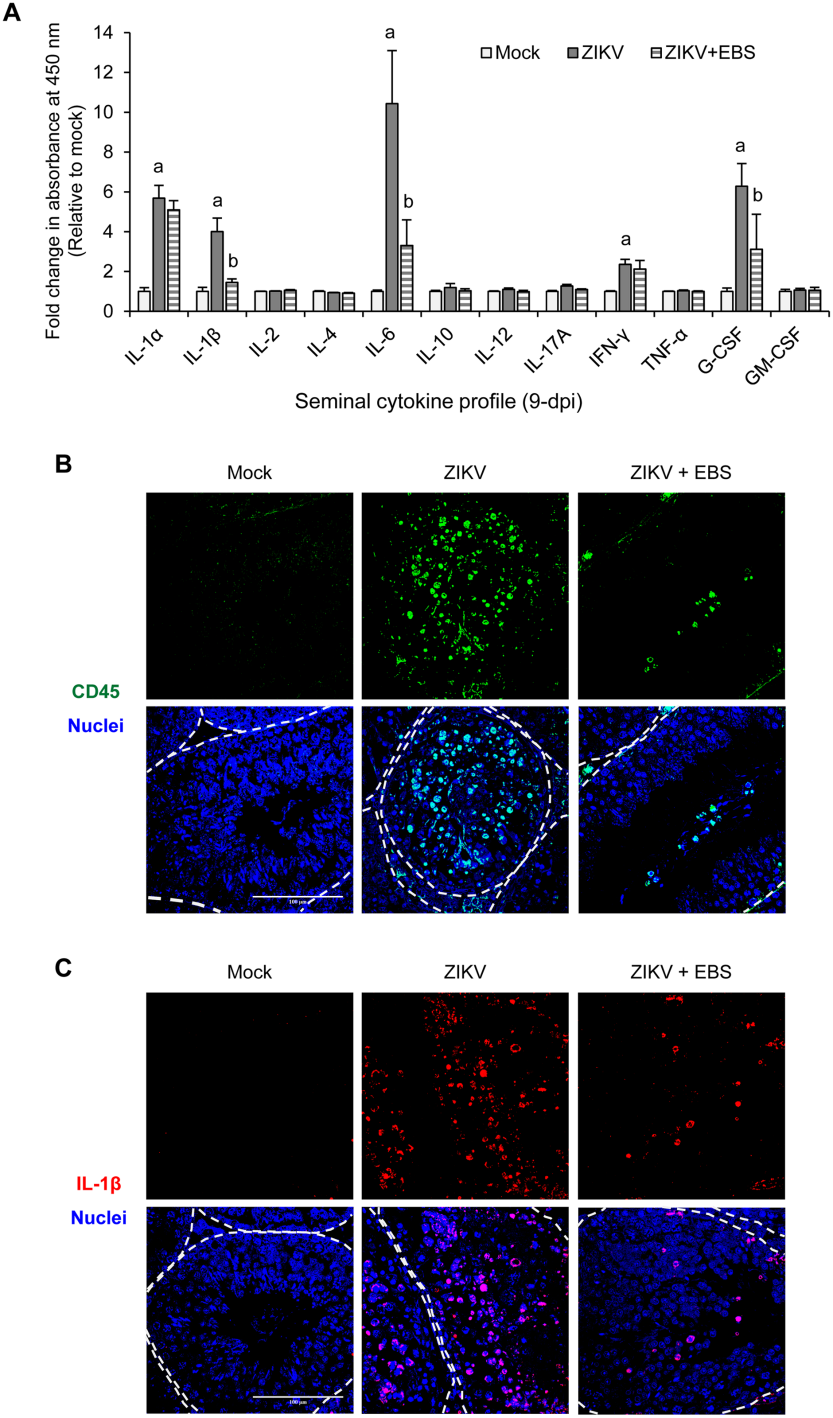
EBS reduces testicular inflammatory response and leucocyte infiltration. AG129 mice subcutaneously infected in the footpad with 5x10^4^ PFU/mouse of ZIKV were intraperitoneally treated with EBS (10 mg/kg body weight/mouse/day) or solvent control on day 1–6 after infection. Testes and sperm were collected on 9 dpi. (A) Cytokine profile of seminal fluid on 9 dpi. Cytokine levels were evaluated by ELISA array. Data are mean ± standard deviation (n = 5 mice/group). A two-tailed 0.01 significance level; a (Mock vs. ZIKV) and b (ZIKV vs. ZIKV+EBS) by Kruskal-Wallis, Bonferroni Post Hoc Test. (B-C) Confocal microscopy of testes sections immunostained for CD45 (green, B), IL-1β (red, C), and Hoechst for nuclei (blue). Scale bar: 100 μm.

We further assessed testicular inflammatory response because high level of ROS may act as signaling molecules to provoke up-regulation of pro-inflammatory cytokines [36]. On 9 dpi, ZIKV significantly increased the production of seminal inflammatory cytokines including IL-1α, IL-1β, IL-6, IFN-γ and G-CSF (Fig 3A; white vs. gray bar). EBS treatment significantly reduced the levels of ZIKV-induced pro-inflammatory cytokines particularly IL-1β, IL-6 and G-CSF (Fig 3A, gray vs. striped bar). In addition, a massive infiltration of CD45^+^ and IL-1β^+^ cells was observed in the SNT of ZIKV-infected testes (Fig 3B and 3C), while EBS treatment reduced the testicular infiltration of CD45^+^ and IL-1β^+^ cells (Fig 3B and 3C). Taken together, ZIKV infection increases testicular oxidative stress and inflammatory response, which can be alleviated by treatment with the antioxidant EBS.

### Antioxidant EBS alleviates ZIKV-associated testicular pathology

**Fig 4:**
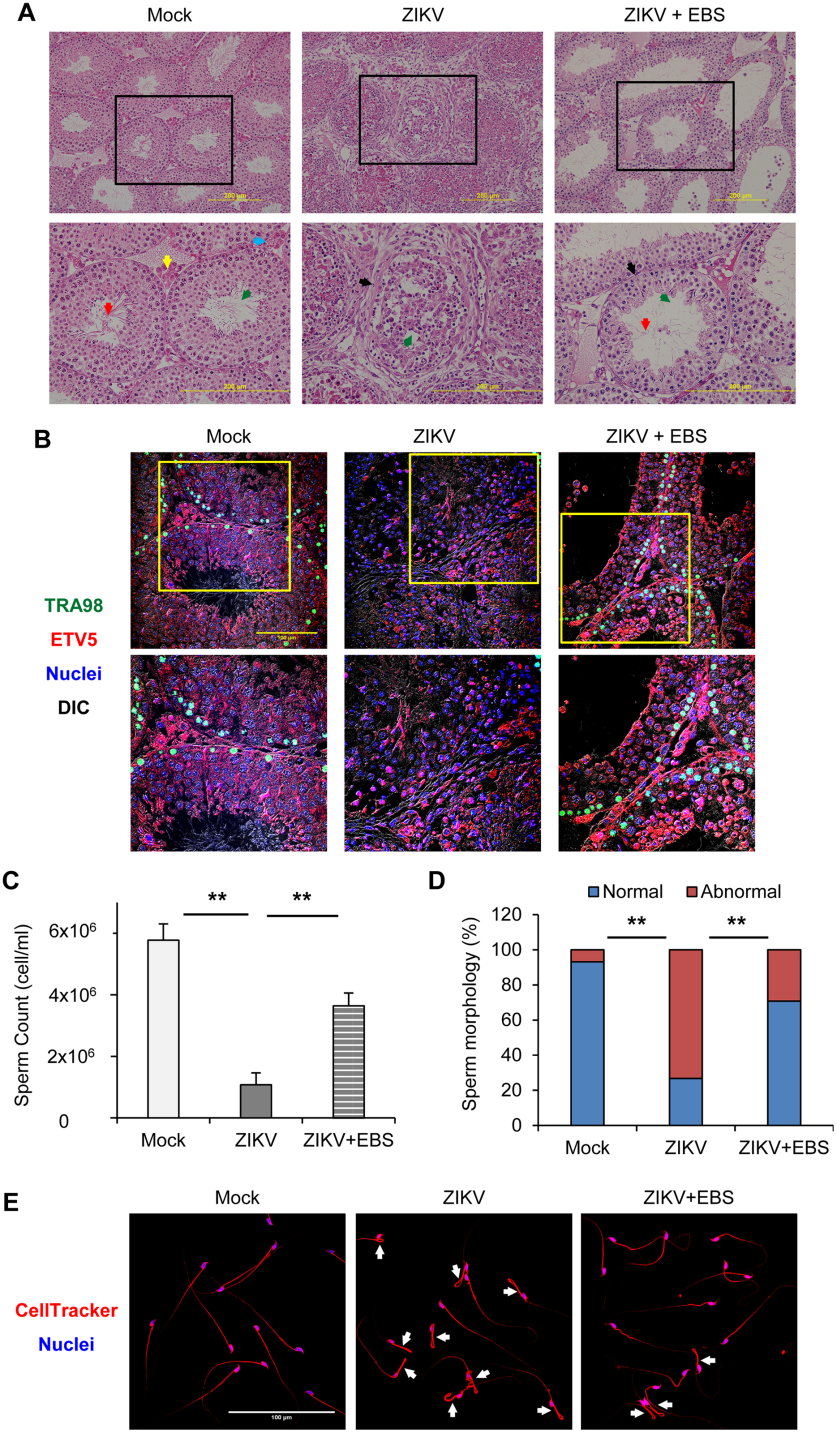
Antioxidant EBS alleviates ZIKV-associated testicular pathology. AG129 mice subcutaneously infected in the footpad with 5x10^4^ PFU/mouse of ZIKV were intraperitoneally treated with EBS (10 mg/kg body weight/mouse/day) or solvent control on 1–6 dpi. Testes and semen were collected on 9 dpi. (A) Histology of testes sections stained with haematoxylin and eosin. Arrows indicate lumen (green), sperm (red), blood capillary (blue), interstitial cell (yellow), and degeneration of SNT (black). Scale bar: 200 μm. (B) Confocal microscopy of testes sections immunostained for TRA98 (green, germ cells), ETV5 (red, blood—testis barrier), and Hoechst for nuclei (blue). Differential interference contrast (DIC). Scale bar: 100 μm. (C) Total number of sperm (cell/ml). (D) Proportion of sperm with normal and abnormal morphology. Data are mean ± SD (n = 6 mice/group). **P<0.01 by Kruskal-Wallis, Bonferroni post-hoc test. (E) Confocal microscopy of sperm morphology with CellTracker staining for cytoplasm (red) and Hoechst for nuclei (blue). Scale bar: 100 μm. Arrows indicate sperm with abnormal morphology.

The role of ROS and pro-inflammatory cytokines in tissue injury has been noted [37]. So, we evaluated whether the inhibitory effect of EBS on testicular oxidative stress and inflammatory response may affect testicular pathology of ZIKV-infected mice on 9 dpi. The testes of control mice showed a regular structure of SNTs, with normal germinal epithelium and accumulation of sperm in the lumen (Fig 4A). In contrast, ZIKV-infected mice showed involution of SNTs, with degeneration of spermatogenic cells in the lumen (Fig 4A). Notably, ZIKV-infected mice receiving EBS treatment during 1–6 dpi displayed a minor pathological feature of SNTs, with few degenerated spermatogenic cells in the lumen (Fig 4A).

In addition, the expression of TRA98 and ETV5 was lower in ZIKV-infected than control testes (Fig 4B). The expression of TRA98 and ETV5 was improved in ZIKV-infected mice receiving EBS versus infected mice receiving solvent (Fig 4B). Sperm parameters were also significantly improved with EBS treatment. Total sperm count was more than three-fold higher in ZIKV-infected mice receiving EBS than those receiving solvent (Fig 4C). This improvement was accompanied by a better sperm morphology profile. EBS- and solvent-treated mice showed about 30% and 70% sperm with abnormal morphology, respectively (Fig 4D and 4E). Hence, EBS treatment attenuated the ZIKV-induced testicular pathology.

### Antioxidant EBS improves testicular oxidative stress, cytokine profile, and pathology of ZIKV-infected C57BL/6 mice

**Fig 5:**
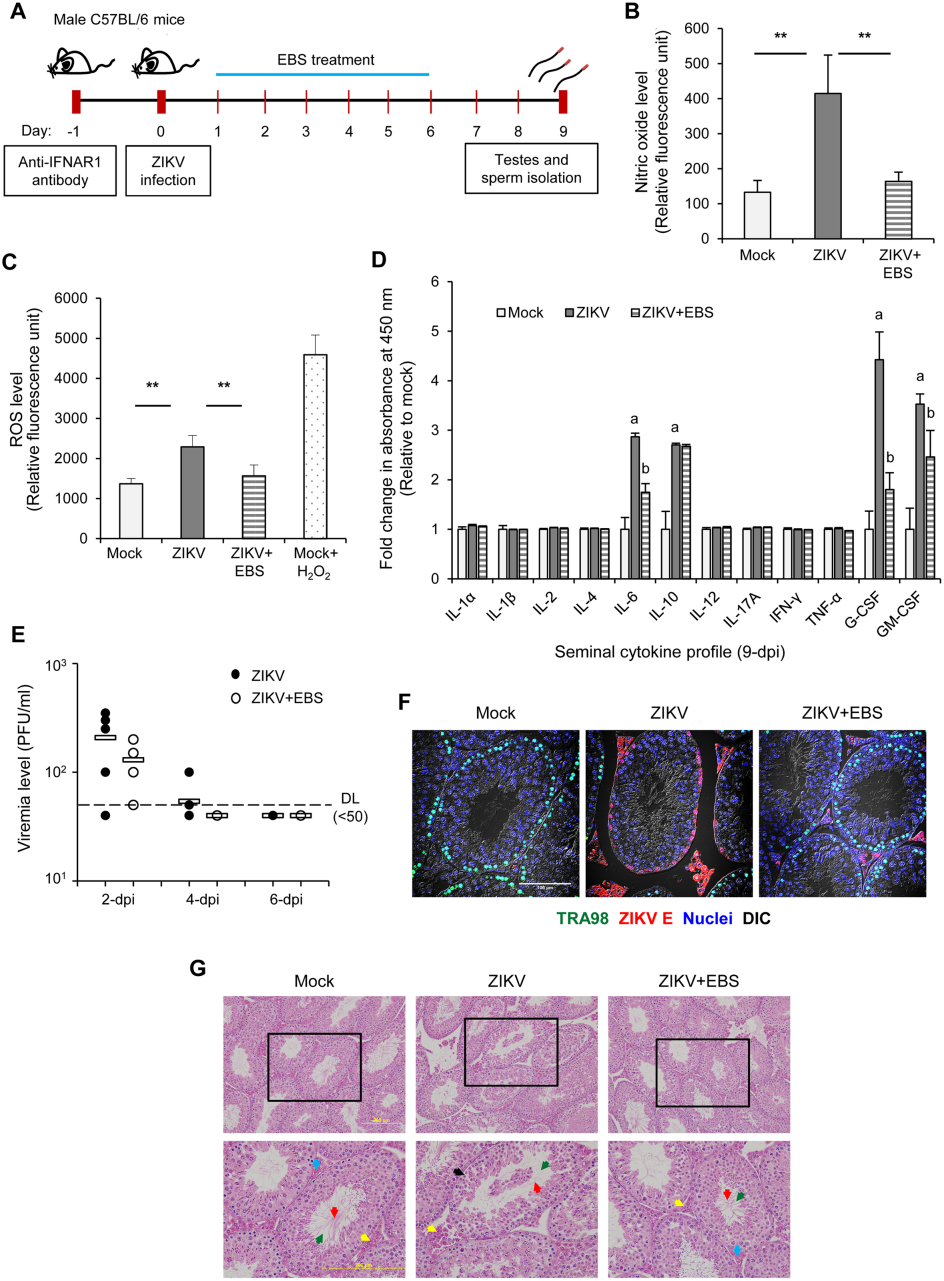
EBS improves oxidative stress, cytokine profile, and testicular pathology of ZIKV-infected C57BL/6 mice. (A) Schematic experimental design. C57BL/6 mice were pre-treated with purified anti-mouse IFNAR-1 antibody (1 mg/kg body weight/mouse/intraperitoneal) one day prior subcutaneous infection of ZIKV (1x10^5^ PFU/mouse) in the footpad. Mice were intraperitoneally treated with EBS (10 mg/kg body weight/mouse/day) or solvent control on 1–6 dpi. Testes and sperm were collected on 9 dpi. (B-C) Intracellular nitric oxide (NO) and reactive oxygen species (ROS) assays. NO (B) and ROS (C) levels in sperms were measured by use of the OxiSelect intracellular NO and ROS indicator, respectively. H_2_O_2_-treated sperms as a positive control. Relative fluorescence intensity was determined by use of a fluorescence plate reader. (D) Cytokine profile of seminal fluid on 9 dpi. Cytokine levels were evaluated by ELISA array. (E) Viremia level on the indicated day after infection. Data are mean ± standard deviation (n = 5 mice/group). *P<0.05 and **P<0.01; a (Mock vs. ZIKV) and b (ZIKV vs. ZIKV+EBS) by Kruskal-Wallis, Bonferroni Post Hoc Test. (F) Confocal microscopy of testes sections immunostained for TRA98 (green, germ cells), ZIKV-E (red), and Hoechst for nuclei (blue). Differential interference contrast (DIC). Scale bar: 100 μm. (G) Histological analysis of testis sections stained with haematoxylin and eosin. Arrows indicate lumen (green), sperm (red), blood capillary (blue), interstitial cell (yellow), and degeneration of SNT (black). Scale bar: 200 μm.

To address the impact of lacking interferon signaling on our findings with AG129 mice, we further performed ZIKV infection study by use of wild-type C57BL/6 mice with anti-IFNAR1 antibody pretreatment as outlined in Fig 5A. ZIKV infection significantly increased NO and ROS levels in sperm and both of them can be reduced by treatment with EBS after ZIKV challenge (Fig 5B and 5C). In addition, ZIKV infection significantly induced seminal inflammatory cytokines including IL-6, IL-10, G-CSF and GM-CSF (Fig 5D; ZIKV vs. Mock). Treatment with EBS repressed the levels of inflammatory cytokines particularly IL-6, G-CSF and GM-CSF (Fig 5D; ZIKV vs. ZIKV+EBS). Slight variation of seminal cytokine profile was noted between C57BL/6 and AG129 mouse models, probably due to the difference on genetic background and/or susceptibility to ZIKV. Viremia could be detected on 2 dpi and EBS treatment slightly reduced the viremia level (Fig 5E). Moreover, ZIKV-E was detected in interstitial cells and primary layer of SNT where germ and sertoli cells are resided (Fig 5F; ZIKV), whereas EBS treatment greatly limited the expression of ZIKV-E in the interstitial cells (Fig 5F; ZIKV+EBS). The expression of ZIKV-E in SNT was accompanied with the downregulation of TRA98 (Fig 5F; ZIKV), while TRA98 remained largely unaffected in EBS-treated mice (Fig 5F; ZIKV+EBS). ZIKV infection also caused degeneration of spermatogenic cells in the lumen of SNT (Fig 5G; ZIKV). In contrast, testis of EBS-treated mice displayed a relatively normal histological structure (Fig 5G; ZIKV+EBS). Taken together, ZIKV-induced testicular oxidative stress, inflammatory response, and pathology noted in the wild-type C57BL/6 mice can also be alleviated by EBS treatment.

### Antioxidant EBS displays a minor effect on ZIKV replication *in vitro* and ZIKV-induced lethality *in vivo*

**Fig 6:**
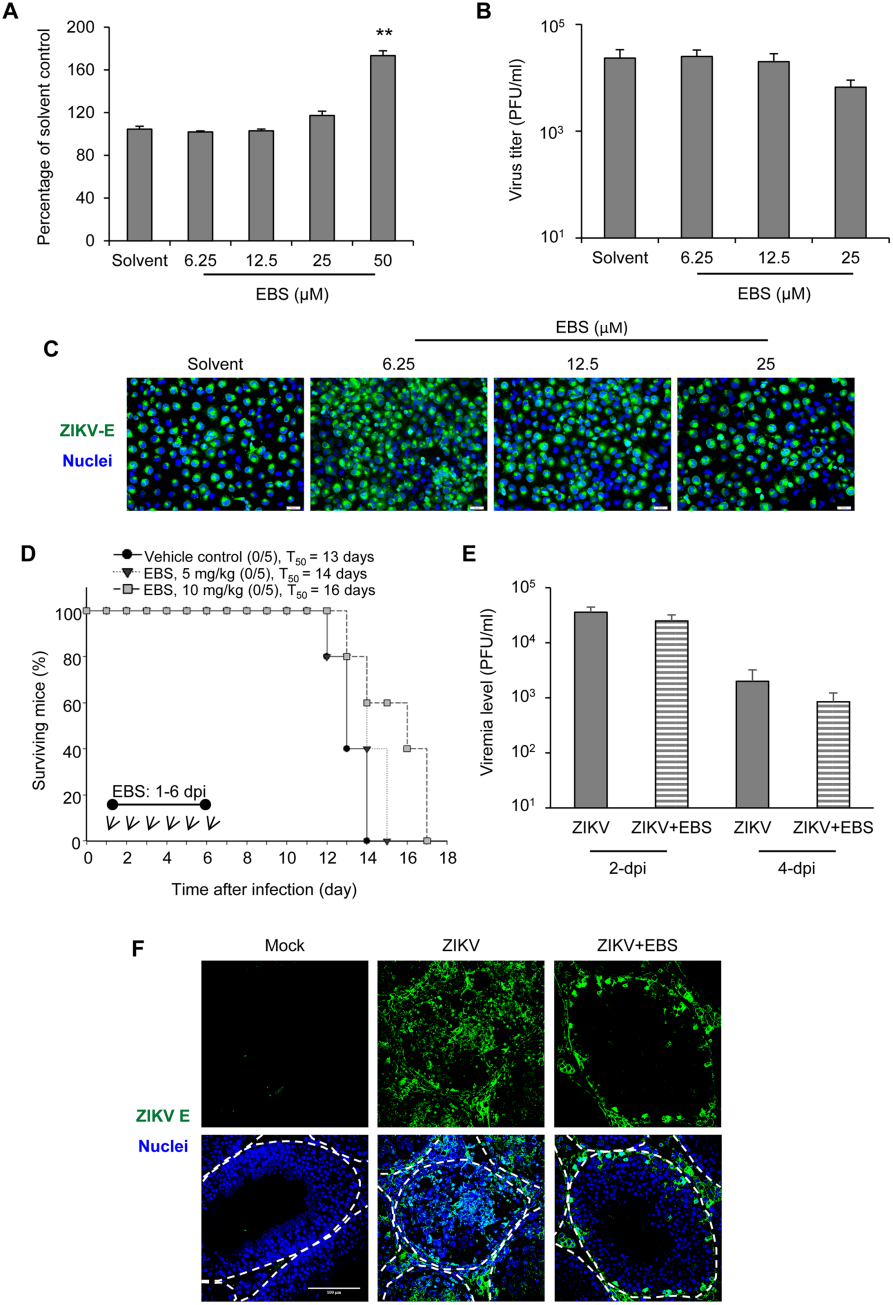
The effects of EBS treatment on viral replication in vitro and ZIKV-induced lethality *in vivo*. (A) EBS cytotoxicity assay. Human microglial CHME3 cells were treated with solvent or the indicated concentrations of EBS for 24 hours. LDH assays was performed to determine cellular cytotoxicity. Data are mean ± SD (n = 5 mice/group or 3 independent experiments). **P < 0.01 by Kruskal-Wallis, Bonferroni post-hoc test. (B-C) Human microglial CHME3 cells were infected with ZIKV (Multiplicity of infection: 0.1) in the presence or absence of EBS for 24 hours. (B) Plaque-forming assay of viral progeny production in culture supernatants. (C) Immunofluorescence microscopy was performed on cells immunostained for ZIKV-E (green) and Hoechst for nuclei (blue). (D-F) AG129 mice were subcutaneously infected in the footpad with 5x10^4^ PFU/mouse of ZIKV. Mice were intraperitoneally treated with EBS (5 or 10 mg/kg body weight/mouse/day) or solvent control on 1–6 dpi marked by arrows. Mice survival was presented as percentage of survival. The median survival time (T_50_) is presented. Survival curves were compared by the use of Log-rank test (Vehicle control vs. EBS 5 mg, P = 0.1327 and Vehicle control vs. EBS 10 mg, P = 0.0505). (E) Viremia level of mice receiving EBS (10 mg/kg body weight) or solvent control. (F) Testicular distribution of ZIKV in mice receiving EBS (10 mg/kg body weight) or solvent control. Confocal microscopy of testes sections immunostained for ZIKV-E (green) and Hoechst for nuclei (blue). Scale bar: 100 μm.

To address whether the protective effect of EBS on testicular pathology is associated with its antiviral potential against ZIKV, we evaluated the anti-ZIKV activity of EBS. First, we determined the non-cytotoxic doses of EBS with lactate dehydrogenase (LDH) release assay on human microglial CHME3 cells. Treatment with EBS up to 25 μM had no cytotoxicity (Fig 6A), but at a higher dose (50 μM) EBS displayed significant cytotoxicity on CHME3 as reported earlier on human hepatoma HepG2 cells [38]. Minor reduction of ZIKV progeny production and viral E protein expression was noted in cells treated with non-cytotoxic 25 μM EBS (Fig 6B and 6C). Furthermore, in AG129 mice challenged with ZIKV, treatment with EBS on 1–6 dpi displayed a minor effect on overall animal survival and viremia level (Fig 6D and 6E). However, EBS treatment greatly limited the expression of ZIKV-E in testes as compared with solvent control, which showed a disperse expression of ZIKV-E in interstitial and spermatogenic cells (Fig 6F). Moreover, as analyzed by western blotting, EBS treatment reduced the expression of ZIKV-E in testis (2.2 fold reduction) and brain (1.4 fold reduction), but not in the spleen (S4 Fig). Thus, EBS exhibited a weak anti-ZIKV activity in culture cells and a tissue-specific antiviral activity in challenged mice. Therefore, the protective effect of EBS on testicular pathology is predominantly associated with its property to reduce testicular oxidative stress and inflammatory response.

### Antioxidant EBS prevents sexual transmission of ZIKV

**Fig 7:**
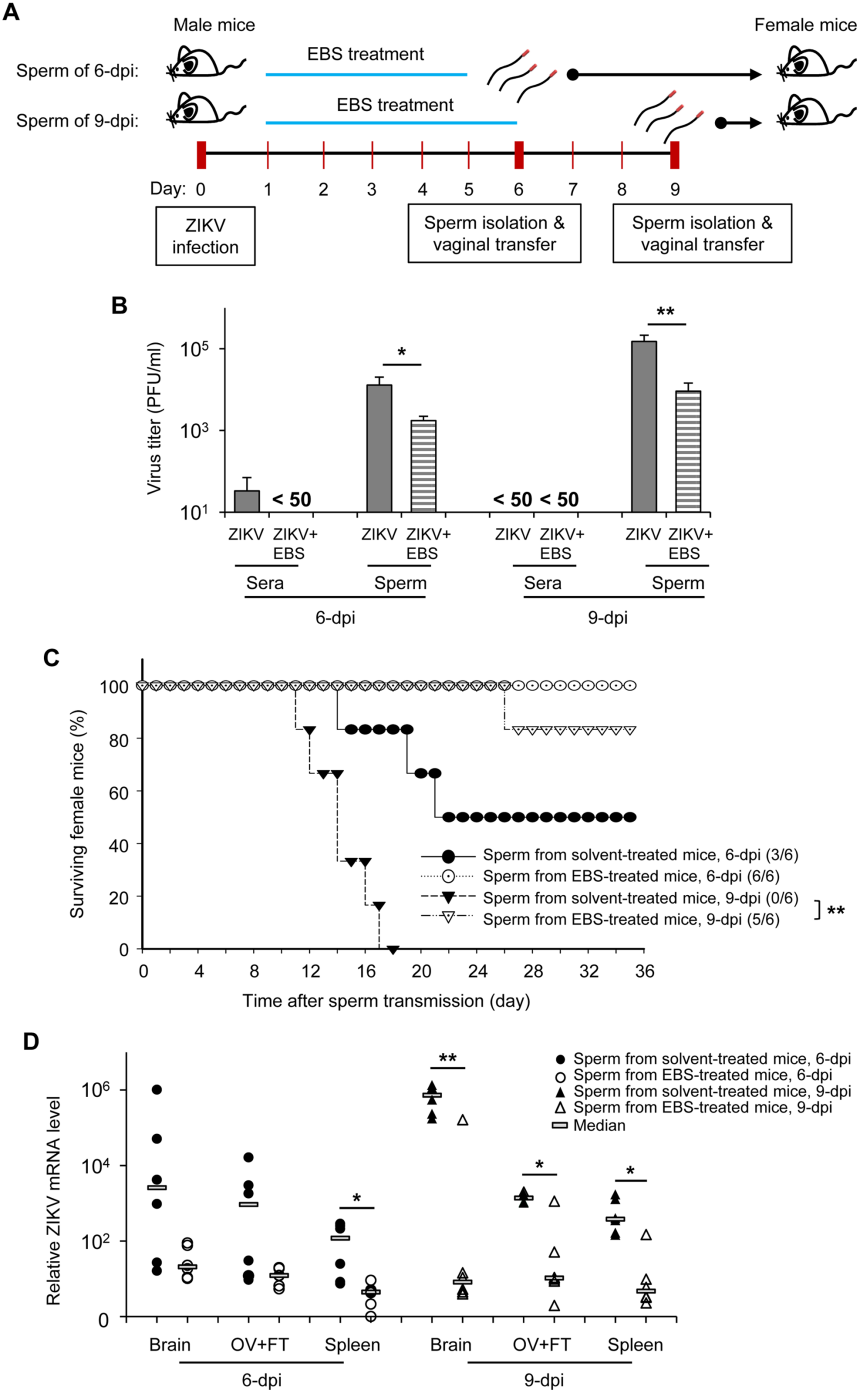
Antioxidant EBS prevents sexual transmission of ZIKV. (A) Schematic experimental design of sexual transmission. AG129 mice subcutaneously infected in the footpad with 5x10^4^ PFU/mouse of ZIKV were intraperitoneally treated with EBS (10 mg/kg body weight/mouse/day) or solvent control on day 1–5 (group: 6-dpi), or day 1–6 (group: 9-dpi) after infection. Sperm and sera were collected on 6 and 9 dpi. Sperm (50 μl) was used for vaginal inoculation into female mice (1 male to 1 female mouse). (B) Plaque-forming assay of viral load in sperm and sera. (C) Survival of female mice receiving sperm. (D) Relative quantitative analysis of ZIKV RNA in brain, ovaries and fallopian tubes (OV+FT), and spleen of recipient female mice. Data are mean ± SD (n = 6 mice/group). *P<0.05 and **P<0.01 by Mann-Whitney U test. Survival curves of female mice were compared by Log-rank test (**P = 0.0005).

We then examined whether EBS treatment may also prevent sexual transmission of ZIKV. Animal models for studying sexual infection of ZIKV have been reported [39–41]. Here, we performed sperm—vaginal transfer from one male to one female mouse as outlined in Fig 7A. Briefly, 50 μl of sperm sample was collected from EBS-treated or solvent-treated ZIKV-infected mice at the indicated time and used for vaginal inoculation into female mice by use of a bent, blunt-end 22-gauge needle to avoid uterine injury. Before this experiment, we evaluated the effect of EBS treatment on ZIKV level in sperm by plaque-forming assay. Treatment with EBS significantly reduced the ZIKV level in sperm as compared with solvent treatment on 6 dpi (Fig 7B; 6-dpi). Although EBS treatment was stopped on 6 dpi, the level of ZIKV in sperm remained significantly lower than in controls on 9 dpi (Fig 7B; 9-dpi).

Next, we used this infectious sperm to challenge female mice by vaginal inoculation. The survival of female mice receiving sperm from solvent-treated mice collected on 6 dpi was 50%, whereas no female mice receiving sperm from EBS-treated mice died (Fig 7C; black vs. empty circle). Sperm from solvent-treated mice collected on 9 dpi led to 0% survival of recipient female mice, whereas 83.33% of female mice receiving sperm from EBS-treated mice survived (Fig 7C; black vs. empty triangle). High viremia was observed in all of the female mice receiving sperm from solvent-treated mice. In contrast, viremia could only be detected in one of the female mice receiving sperm from EBS-treated mice (S5 Fig), well correlating with the survival data.

We also tested the efficacy of other antioxidants, vitamin C and quercetin, to improve testicular pathology and prevent sexual transmission of ZIKV (S6 Fig). Vitamin C and quercetin are the most abundant antioxidants found in dietary sources [42]. Treatment with vitamin C and quercetin did not affect the overall survival of recipient female mice. However, the median survival time (T_50_) of female mice receiving sperm from vitamin C-treated mice was significantly improved than those receiving sperm from solvent-treated mice (S6D Fig; black vs. empty circle; T_50_ = 13.5 vs. 17.5 days; P = 0.011). The T_50_ did not differ between female mice receiving sperm from quercetin-treated mice and controls (S6D Fig; black circle vs. triangle; T_50_ = 13.5 vs. 12 days; P = 0.677). These data suggest that different antioxidants may have different efficacy in preventing the sexual transmission of ZIKV. Finally, we examined tissue from female mice receiving sperm transfer to confirm the systemic infection of ZIKV. RNA of ZIKV was detected in brain, ovary-fallopian tubes, and spleen (Fig 7D). Collectively, these data suggest that EBS treatment may prevent seminal transmission of ZIKV.

## Discussion

Here we show the disease progression of ZIKV in the testicular tissue of AG129 mice lacking interferon receptors and also in wild-type C57BL/6 mice pretreated with anti-IFNAR-1 antibody; both animal models are useful for studying ZIKV replication, tropism, immunity, and transmission [41, 43, 44]. In addition, we developed an animal model to study male-to-female sexual transmission of ZIKV and identified a possible therapeutic intervention to minimize the transmission. ZIKV progressively infects testis by gaining access to interstitial cells, the basement membrane of the SNT, spermatogenic cells, and sperm. ZIKV infection leads to several testicular pathologies, including involution of the SNT, degeneration of spermatogenic cells, oligospermia, a high proportion of sperm with abnormal morphology, poor expression of TRA98 and ETV5. In agreement with a previous study [14], our data suggest that ZIKV infection may cause male infertility. Moreover, ZIKV infection increased ROS levels and impaired cellular antioxidant of sperm. ROS is considered a risk factor for some diseases including cancer and infection [45, 46]. Our data indicate that high ROS levels may be associated with high level of seminal pro-inflammatory cytokines, poor testicular outcome and high level of infectious ZIKV in sperm. ROS derived as a byproduct of viral replication can accelerate pro-inflammatory response to cause endothelial barrier disruption and tissue injury [15, 37]. Thus, oxidative system may be one of the cellular factors attributed to testicular pathogenesis and sexual transmission of ZIKV. In testicular dysfunction, reducing ROS levels is a common therapeutic strategy to improve the disease prognosis [17, 18]. We used this strategy to alleviate testicular pathogenesis and prevent seminal transmission of ZIKV.

Sexual transmission of ZIKV could be as critical as mosquito-borne transmission in the context of maternal infection. By August 2016, the World Health Organization had documented the sexual transmission of ZIKV in 11 countries worldwide [12]. The presence of infectious ZIKV in the semen poses a potential risk of sexual transmission. However, one may argue a sustainable transmission of ZIKV via sexual intercourse [47]. Our data indicate that seminal ROS and inflammatory cytokines may contribute to successful sexual transmission of ZIKV. Despite the substantial level of ZIKV detected in the sperm of EBS-treated mice on 6 dpi (Fig 7B; ZIKV+EBS, 6-dpi), the sperm failed to establish ZIKV transmission in female mice (Fig 7C; empty circle). Furthermore, the sperm of EBS-treated mice on 9 dpi caused only 16.67% mortality in female mice, although the ZIKV level in sperm did not greatly differ from that of solvent-treated mice on 6 dpi, which killed 50% of the transmitted female mice (Fig 7B; ZIKV+EBS [9-dpi] vs. ZIKV [6-dpi], P = 0.37). This finding can be explained in part by a relatively lower level of seminal ROS in EBS- than solvent-treated mice (Fig 2B; ZIKV+EBS [9-dpi] vs. ZIKV [6-dpi], P = 0.03). Correlation study and regression modeling are warranted to determine whether the level of ROS and inflammatory cytokines can be a predictor of successful sexually transmitted ZIKV. It has recently become apparent that semen acts directly on tissues in the female reproductive tract. Indeed, a substantial level of seminal pro-inflammatory cytokines and ROS may provoke inflammatory responses in the female reproductive tract and cause tissue injury [37, 48]. Consequently, these processes may create access to and favorable conditions for seminal infectious ZIKV to establish an initial infection in the female reproductive tract before its systemic infection.

EBS displays a weak effect on ZIKV replication in culture cells and ZIKV-induced lethality in challenged animals. However, a tissue-specific restriction on ZIKV was noted in the infected mice. We speculate that the protective effect of EBS on testicular pathology and sexual transmission of ZIKV is associated with its efficacy to reduce the level of oxidative stress and pro-inflammatory cytokine in testes. EBS, a synthetic antioxidant, has a unique characteristic as compared with the other antioxidants. EBS may reduce the production of NO by inhibiting the catalytic activity of nitric oxide synthases (NOS) [23]. Consequently, EBS may also suppress the level of NO-associated inflammatory cytokines [49]. Recently, EBS has been demonstrated to reduce the level of multidrug-resistant staphylococcal-associated inflammatory cytokines including IL-1β and IL-6 [50]. A substantial level of NO activates IL-6 production through MAPK signaling pathway and increased IL-6 mRNA stability [24]. In addition, the protective effects of EBS against oxidative stress rely on its GPx-like activity [19]. It is an effective scavenger of lipid hydroperoxides mimicking the action of GPx4 [21]. GPx4 is one of the most important GPx isoforms in a testicular context and is abundantly expressed in spermatogenic cells, particularly sperm [22, 51]. Therefore, EBS may have beneficial properties for the treatment of testicular pathology while more effective testis-specific drugs or antioxidants remain to be discovered. Our data indicate that EBS also displays efficacy to reduce ZIKV load in brain. Interestingly, GPx4 plays a role in brain development and neuronal apoptosis [52]. GPx4 deficient mice die in utero at midgestation with a major defect in embryonic brain development [53]. The implication of GPx4 in female reproduction remains unknown and further studies to evaluate whether EBS may alleviate ZIKV pathogenesis in female reproductive system are warranted.

Antioxidants have been used to improve the prognosis of viral diseases. In a case report, high doses of intravenous vitamin C given over 2 days may have resolved symptoms of a patient with Chikungunya [54]. In addition, vitamin C given over 3 days in increment doses up to 75 g substantially improved symptoms of a patient with ZIKV infection [55]. The mechanism of action of antioxidant vitamin C remains controversial. However, studies suggest that vitamin C reduces inflammatory responses and ROS levels in a glutathione-independent mechanism [56, 57]. Similarly, a flavonolic antioxidant, quercetin, displays its antioxidant properties in part by suppressing the inflammatory response and scavenging peroxyl radicals [58, 59]. However, we did not see a potential use of quercetin (10 mg/kg bw) to prevent sexual transmission of ZIKV. We speculate that the efficacy of antioxidants to prevent sexual transmission of ZIKV may vary.

The dosage and treatment duration of antioxidant as a therapeutic agent must be carefully interpreted. Antioxidants have beneficial effects at a physiologic dose. However, at a relatively high dose, they may have unfavorable effects [42]. The dosage of 10 mg EBS/kg bw/day for mice is equivalent to 0.811 mg/kg bw/day for humans, as calculated by a published method [60]; thus, for a 60-kg person, the dosage would be 48.6 mg/day. We used the treatment duration of EBS of 6 days because viremia can still be detected on 6 dpi. EBS is being used in clinical trials for various diseases [19]. A study to evaluate the safety and pharmacokinetics of 200 mg EBS oral capsule (SPI-1005) in healthy adults has been completed (NCT01452607). Therefore, the present study strongly argues for a potential clinical use of EBS to alleviate the testicular pathogenesis with ZIKV infection and its sexual transmission.

## Materials and methods

### Ethics statement

The mouse experiments were conducted according to the guideline outlined by Council of Agriculture Executive Yuan, Republic of China. This animal protocol was approved by the Academia Sinica Institutional Animal Care and Use Committee (Protocol no. 16-06-966) and were performed in accordance with the guidelines. Infection was performed in mice under isoflurane anesthesia and all efforts were made to minimize animal suffering.

### Mouse infection experiments

Five-week-old male AG129 mice with interferon-α/β and -γ receptor knockout were subcutaneously infected in the footpad with 5×10^4^ plaque-forming units (PFU) of ZIKV per mouse. Testes, sperm, and sera were collected on 3, 6, and 9 days post-infection (dpi). Five-week-old male C57BL/6 mice were pre-treated with purified anti-mouse IFNAR-1 antibody (1 mg/kg body weight/mouse/intraperitoneal), one day prior to subcutaneous inoculation of ZIKV (1×10^5^ PFU/mouse) in the footpad. Testes, sperm, and sera were collected on 9 dpi. To study the effect of the antioxidant ebselen (EBS, Cayman Chemical, CAS 60940-34-3) on testicular pathology, sexual transmission, and ZIKV-induced lethality, mice were treated with EBS (10 mg/kg body weight/mouse/intraperitoneal/day) or solvent control (phosphate buffered saline [PBS]+10% polyethylene glycol as a co-solvent) on days 1–2 (Group: 3-dpi), 1–5 (Group: 6-dpi), or 1–6 (Group: 9-dpi) after ZIKV infection. To study the effect of the antioxidants vitamin C (L-Ascorbic acid, Sigma, A4403) and quercetin (Alfa Aesar, A15807), mice were treated with vitamin C (Vit C, 10 mg/kg body weight/mouse/intraperitoneal/day), quercetin (Quer, 10 mg/kg body weight/mouse/intraperitoneal/day), or solvent control (PBS) on day 1–6 after infection. To study the sexual transmission of ZIKV, we used a modified protocol of artificial insemination in mice [61]. Estrous cycle may interfere the susceptibility of mice to transgenital transmission of ZIKV [44]. Therefore, four-week-old female AG129 mice were used to minimize the effect of hormonal staging in evaluating the efficacy of EBS. Briefly, 50 μl sperm of one male mouse was transferred into the vagina of one female mouse. A 120°-bent, blunt-end 22-gauge needle (Taiwan Vacuum Technology, D6622-45) was used for vaginal inoculation to avoid uterine injury. The mice were checked daily for severe symptoms including limb paralysis. Euthanasia of mice with severe limb paralysis was performed as the endpoint of animal survival. The brain, spleen, ovaries and fallopian tubes were immediately isolated after the animal died for estimating ZIKV RNA level.

### Virus propagation, plaque-forming assay, and quantitative RT-PCR (qRT-PCR)

ZIKV PRVABC59 strain (2015 Puerto Rico strain, Genbank accession: KU501215) was kindly provided by the Centers for Disease Control, Taiwan. The virus was propagated in C6/36 mosquito cells (ATCC: CRL-1660) and the level of infectious virus particles was measured by plaque-forming assay (PFU/ml) in Vero cells (ATCC: CRL-1587) as described [62]. Total RNA was extracted from homogenized animal tissues by using the RNeasy kit (QIAGEN) and level of ZIKV RNA was analyzed by real-time RT-PCR (Roche LightCycler 2.0). Briefly, cDNA was reverse-transcribed from 1 μg of RNA with random hexamers by using the ThermoScript RT kit (Invitrogen). PCR involved use of the LightCycler FastStart DNA Master PLUS SYBR Green I kit (Roche) with the primers for ZIKV (5′-CCGCTGCCCAACACAAG-3′ and 5′-CCACTAACGTTCTTTTGCAGACAT-3′) and β-actin (5′-TCCTGTGGCATCCACGAAACT-3′ and 5′-GAAGCATTTGCGGTGGACGAT-3′). ZIKV RNA level was normalized to that of actin for relative quantification.

### Sperm isolation and examination

Sperm was isolated as described [61] with modification. To isolate mature sperm, cauda epididymides were placed in 100 μl Vitro Fert medium (Cook Medical, K-RVFE 50) and gently sliced by using an 18-gauge needle. After a few minutes, epididymides were removed and suspension (sperm) was used for evaluating sperm quality, reactive oxygen species (ROS) and nitric oxide (NO) level, cytokine profile, plaque-forming assay, and sexual transmission study.

Sperm quality (sperm count and morphology) was evaluated according to the guidelines of the World Health Organization [28]. Trypan blue staining was performed and sperm number was determined by using a hemocytometer. Air-dried sperm smears were used for morphological and immunostaining assays. To evaluate sperm morphology by microscopy, sperm smears were fixed with 4% paraformaldehyde (PFA) and stained with Hoechst (Invitrogen, H21492) for nuclei and with CellTracker Orange (Invitrogen, C2927) for cytoplasm without permeabilization. For immunofluorescence assay, sperm smears were permeabilized with 0.5% TritonX-100 for 10 min, blocked with 5% skim milk in PBS for 1 hour and immunostained overnight with anti-flavivirus E [63], anti-dsRNA (Scicons, J2), anti-GPx4 (Abcam, Ab125066), or anti-SPATA18 (Abgent, AP13960a) antibodies. Confocal analysis was performed by using confocal laser scanning microscope, ZEISS LSM 700. Fluorescence intensity of confocal images was determined by using ImageJ (US National Institutes of Health). To determine the level of infectious ZIKV, sperm was subjected to five rapid freeze-thaw cycles to release virus particles from sperm before plaque-forming assay.

The level of ROS and NO in sperm was determined by using the OxiSelect Intracellular ROS (Cell Biolabs, STA-342) and NO (Cell Biolabs, STA-800) Assay kit, respectively. The fluorescence probe DCFH-DA was used to measure hydroxyl, peroxyl, or other ROS level within a cell, while NO fluorometric probe was used to measure the level of intracellular NO. Briefly, sperm was plated on 96-well black microplates at 5x10^4^ sperm/well, then incubated with 1X DCFH-DA or 1X NO fluorometric probe (diluted in Hank’s Balanced Salt Solution without phenol red) for 45 min at 37°C. Sperm treated with 500 μM H_2_O_2_ was a positive control. Fluorescence intensity was determined by using a fluorescence reader at 480/530 nm (excitation/emission) [64]. For cytokine assay, sperm samples were centrifuged and supernatants (10 μl) were subjected to mouse inflammatory cytokines multi-analyte ELISArray (Qiagen, MEM-004A).

### Histology, immunohistochemistry, and western blot analysis

Testes were collected immediately after death and fixed overnight in Bouin’s solution (Sigma, HT10132), washed with 50% alcohol and then underwent tissue processing and embedding. Haematoxylin-eosin staining was performed on 3-μm-thick testes sections for histology. For immunohistochemistry, testes sections were immunostained with anti-flavivirus E [63], anti-TRA98 (Abcam, ab82527), anti-ETV5 (Abcam, ab102010), anti-CD45 (Novusbio, NB100-77417), and anti-IL1β (Abcam, ab2105) antibodies. Testis, brain, and spleen were digested in cold RIPA lysis buffer, sonicated at 21% amplitude for 2 min, and then incubated on ice for 30 min. Homogenate was centrifuge at 10,000 rpm for 10 min at 4°C and supernatants were collected for western blot analysis to evaluate the expression of ZIKV-E protein.

### Cell culture study

Human microglial CHME3 cells [65] were grown in DMEM containing 10% fetal bovine serum. Cytotoxicity of EBS was determined by using lactate dehydrogenase (LDH) assay (Roche). Briefly, cells treated with EBS at the indicated doses for overnight underwent LDH assay. Sample absorbance was determined by using an ELISA reader (molecular device) at 490 nm. For antiviral study, cells in 12-well plates were adsorbed with ZIKV at 0.1 of multiplicity of infection for 2 hours with the indicated doses of EBS, washed thoroughly to remove unbound viruses, then incubated for another 24 hours in the presence or absence of EBS. The antiviral effect of EBS was evaluated by immunofluorescence and plaque-forming assays.

### Statistical analysis

Data were compared by Mann-Whitney or Kruskal-Wallis Bonferroni post-hoc test. Statistical significance was set at *P* < 0.05. Survival curves were descriptively analyzed by using SigmaPlot v10.0 (Systat Software) and compared by Log-rank test with use of Prism v5.0 (GraphPad Software).

## Supporting information

S1 Fig ZIKV infects sperm. Confocal microscopy images of pool sperm immunostained for double-stranded dsRNA (green), CellTracker for cytoplasm (red), and Hoechst for nuclei (blue). Scale bar: 100 μm. n = 6 mice/group.
(TIF)

S2 Fig ZIKV damages testes and reduces sperm quality. (A) Histological analysis of testis sections stained with haematoxylin and eosin. Arrows indicate lumen (green), sperm (red), blood capillary (blue), interstitial cell (yellow), and degeneration of SNT (black). Scale bar: 200 μm. (B) Confocal microscopy of testis sections immunostained for TRA98 (green, germ cells), ETV5 (red, blood—testis barrier), and Hoechst for nuclei (blue). Differential interference contrast (DIC). Scale bar: 100 μm. (C) Confocal microscopy of pool sperm stained with CellTracker for cytoplasm (red) and Hoechst for nuclei (blue). Arrows indicate abnormal sperm morphology. Scale bar: 100 μm. n = 6 mice/group.
(TIF)

S3 Fig ZIKV infection increases testicular oxidative stress. (A) Intracellular NO assay. NO level in 9 dpi sperm was measured by use of OxiSelect intracellular NO indicator. Relative fluorescence intensity was determined by use of fluorescence plate reader. Data are mean ± SD (n = 5 mice/group). **P<0.01 by Kruskal-Wallis, Bonferroni post-hoc test. (B) Confocal microscopy of sperm immunostained for GPx4 (green), CellTracker for cytoplasm (red), and Hoechst for nuclei (blue). (C) Confocal microscopy of sperm immunostained for SPATA18 (green), CellTracker for cytoplasm (red), and Hoechst for nuclei (blue). Scale bar: 100 μm.
(TIF)

S4 Fig The effect of EBS on ZIKV load in testes and other tissues. AG129 mice subcutaneously infected in the footpad with 5x10^4^ PFU/mouse of ZIKV were intraperitoneally treated with EBS (10 mg/kg body weight/mouse/day) or solvent control on 1–6 dpi. Testis, brain, and spleen were collected on 9 dpi. Western blot analysis of protein levels of ZIKV-E and β-actin for loading control; Data are ratios of ZIKV-E and β-actin density (n = 5 mice/group). *P<0.05 by Mann-Whitney U test.
(TIF)

S5 Fig Viremia level of female mice receiving sperm from ZIKV-infected mice. AG129 mice subcutaneously infected in the footpad with 5x10^4^ PFU/mouse of ZIKV were intraperitoneally treated with EBS (10 mg/kg body weight/mouse/day) or solvent control on 1–6 dpi. Sperm (50 μl) collected on 9 dpi was used for vaginal inoculation into female AG129 mice (1 male to 1 female mouse). Plaque-forming assay of viral load in sera of female mice on the indicated day after sperm transfer. Data are individual and mean (n = 5 mice/group). **P<0.01 by Mann-Whitney U test.
(TIF)

S6 Fig Effect of other antioxidants on sexual transmission of ZIKV. (A) Histological analysis of testis sections stained with haematoxylin and eosin. Arrows indicate lumen (green), sperm (red), blood capillary (blue), interstitial cell (yellow), and degeneration of SNT (black). Scale bar: 200 μm. (B) Intracellular ROS assay. ROS levels in sperm were measured by use of the OxiSelect intracellular ROS indicator. Relative fluorescence intensity was determined by use of a fluorescence plate reader. (C) Plaque-forming assay of viral load in sperm. (D) Survival of female mice receiving semen transfer. Testes and sperm were collected on day 9 after infection. Data are mean ± SD (n = 6 mice/group). *P<0.05 and **P<0.01 by Kruskal-Wallis, Bonferroni post-hoc test. The median survival time (T_50_) is presented. Survival curves of female mice were compared by Log-rank test (*P = 0.01). Abbreviation: Vit C, vitamin C; Quer, quercetin.
(TIF)
