# Supplementary figures and images for: Ebselen alleviates testicular pathology in mice with Zika virus infection and prevents its sexual transmission
Source: PLoS Pathog. 2018 Feb 15;14(2):e1006854. doi: 10.1371/journal.ppat.1006854 (PMC5814061; doi:10.1371/journal.ppat.1006854)

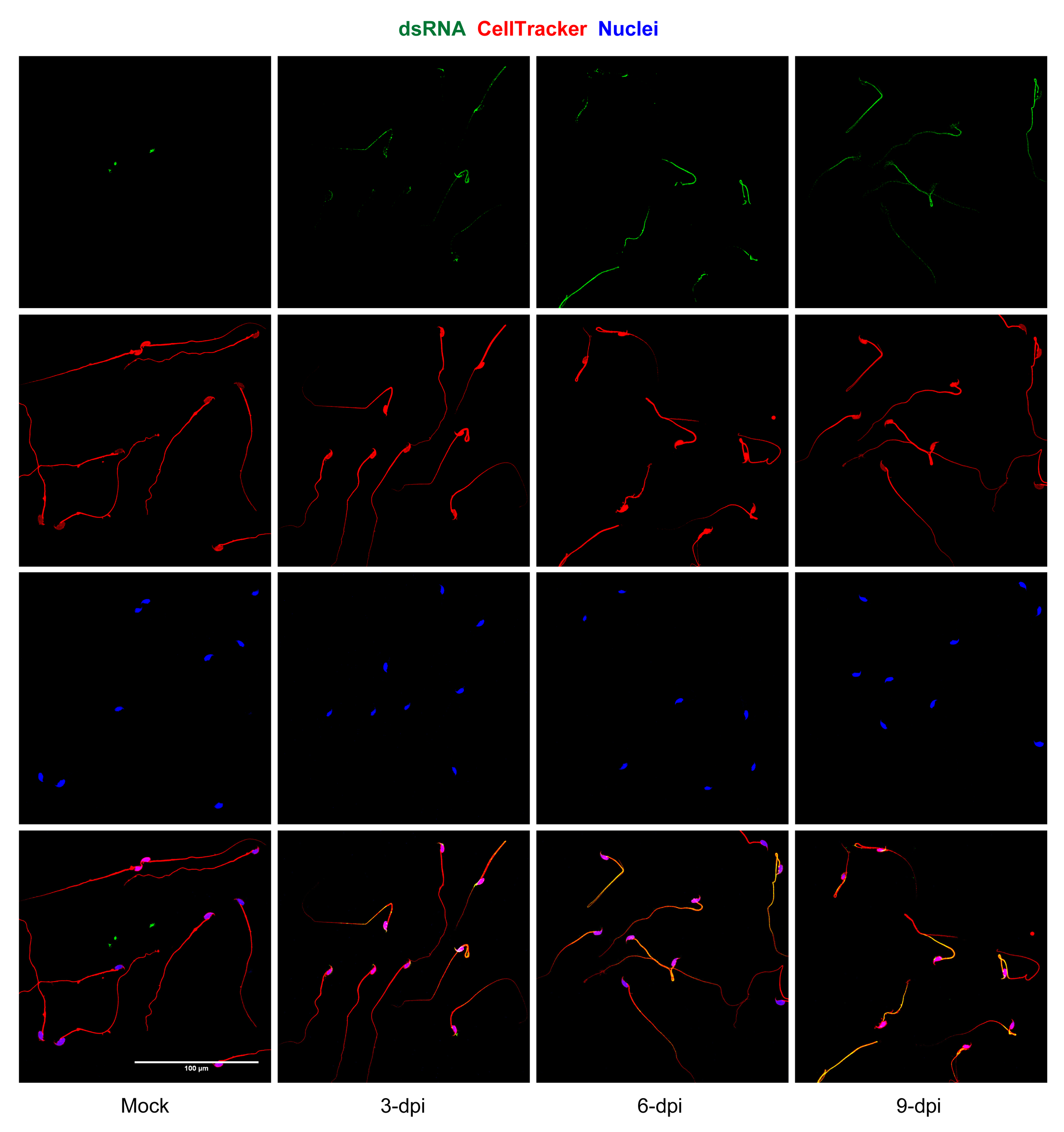

Supplement: S1 Fig — Confocal microscopy images of pool sperm immunostained for double-stranded dsRNA (green), CellTracker for cytoplasm (red), and Hoechst for nuclei (blue). Scale bar: 100 μm. n = 6 mice/group. (TIF) [file ppat.1006854.s001.tif]

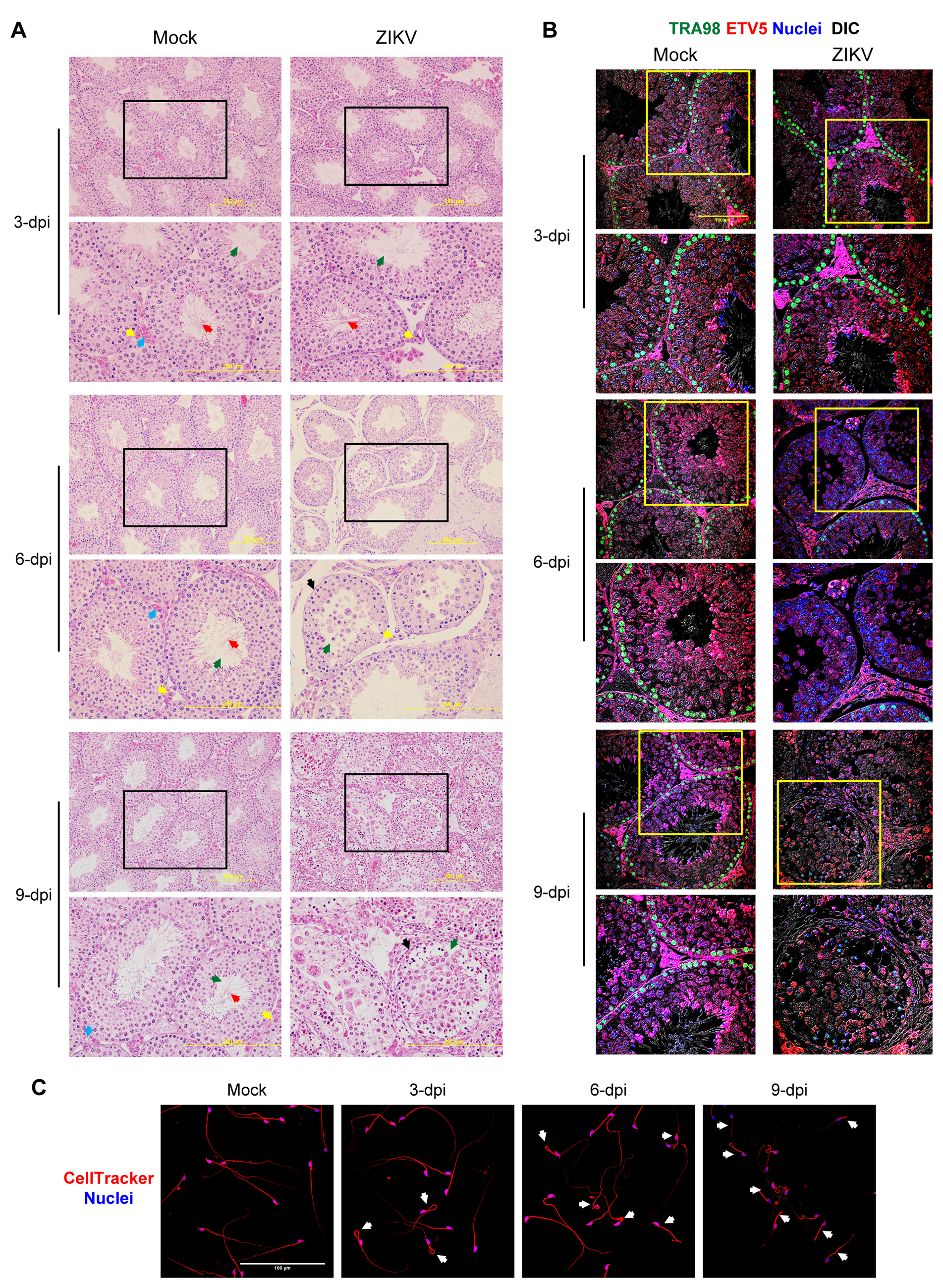

Supplement: S2 Fig — (A) Histological analysis of testis sections stained with haematoxylin and eosin. Arrows indicate lumen (green), sperm (red), blood capillary (blue), interstitial cell (yellow), and degeneration of SNT (black). Scale bar: 200 μm. (B) Confocal microscopy of testis sections immunostained for TRA98 (green, germ cells), ETV5 (red, blood—testis barrier), and Hoechst for nuclei (blue). Differential interference contrast (DIC). Scale bar: 100 μm. (C) Confocal microscopy of pool sperm stained with CellTracker for cytoplasm (red) and Hoechst for nuclei (blue). Arrows indicate abnormal sperm morphology. Scale bar: 100 μm. n = 6 mice/group. (TIF) [file ppat.1006854.s002.tif]

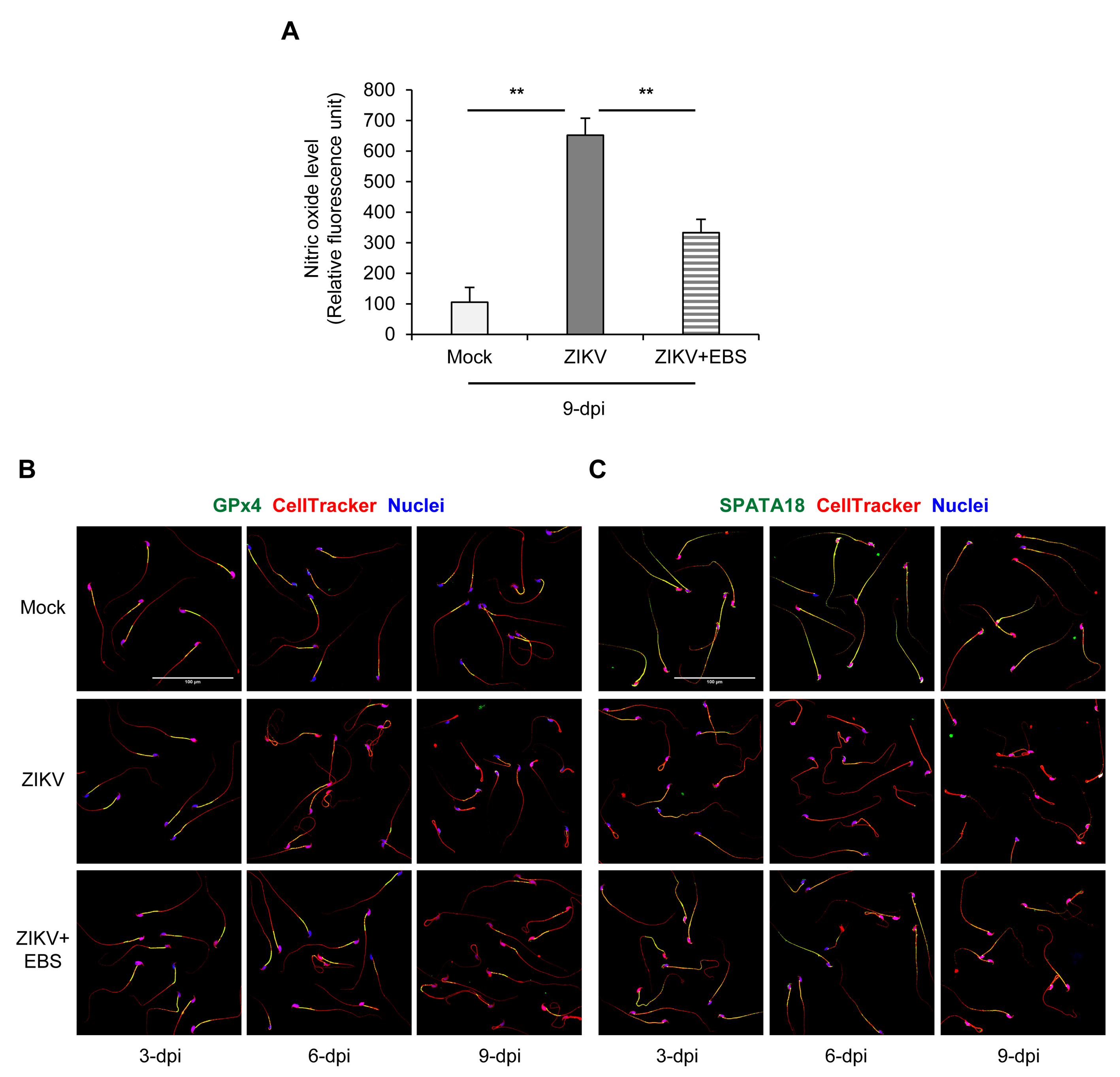

Supplement: S3 Fig — (A) Intracellular NO assay. NO level in 9 dpi sperm was measured by use of OxiSelect intracellular NO indicator. Relative fluorescence intensity was determined by use of fluorescence plate reader. Data are mean ± SD (n = 5 mice/group). **P<0.01 by Kruskal-Wallis, Bonferroni post-hoc test. (B) Confocal microscopy of sperm immunostained for GPx4 (green), CellTracker for cytoplasm (red), and Hoechst for nuclei (blue). (C) Confocal microscopy of sperm immunostained for SPATA18 (green), CellTracker for cytoplasm (red), and Hoechst for nuclei (blue). Scale bar: 100 μm. (TIF) [file ppat.1006854.s003.tif]

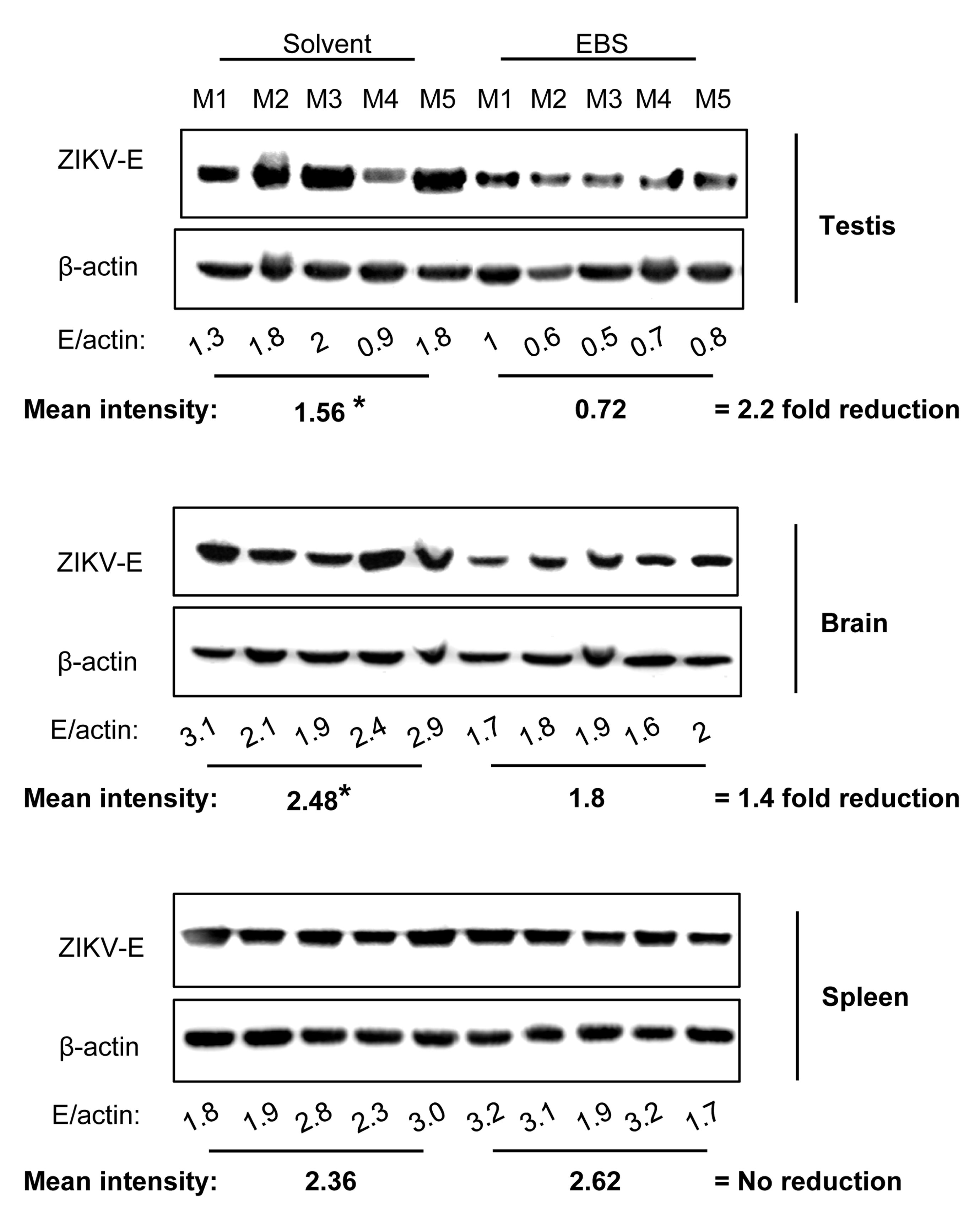

Supplement: S4 Fig — AG129 mice subcutaneously infected in the footpad with 5x10^4^ PFU/mouse of ZIKV were intraperitoneally treated with EBS (10 mg/kg body weight/mouse/day) or solvent control on 1–6 dpi. Testis, brain, and spleen were collected on 9 dpi. Western blot analysis of protein levels of ZIKV-E and β-actin for loading control; Data are ratios of ZIKV-E and β-actin density (n = 5 mice/group). *P<0.05 by Mann-Whitney U test. (TIF) [file ppat.1006854.s004.tif]

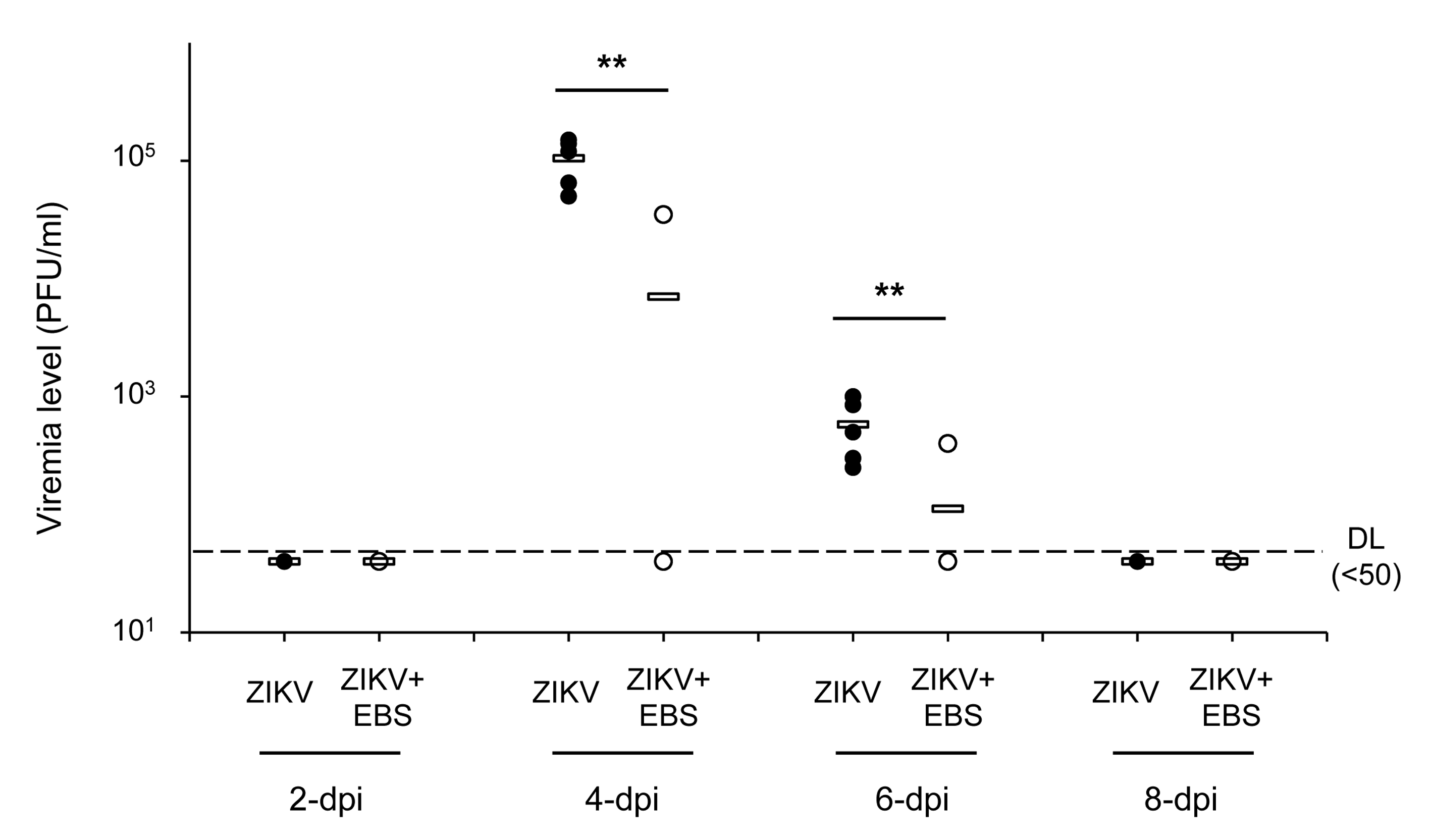

Supplement: S5 Fig — AG129 mice subcutaneously infected in the footpad with 5x10^4^ PFU/mouse of ZIKV were intraperitoneally treated with EBS (10 mg/kg body weight/mouse/day) or solvent control on 1–6 dpi. Sperm (50 μl) collected on 9 dpi was used for vaginal inoculation into female AG129 mice (1 male to 1 female mouse). Plaque-forming assay of viral load in sera of female mice on the indicated day after sperm transfer. Data are individual and mean (n = 5 mice/group). **P<0.01 by Mann-Whitney U test. (TIF) [file ppat.1006854.s005.tif]

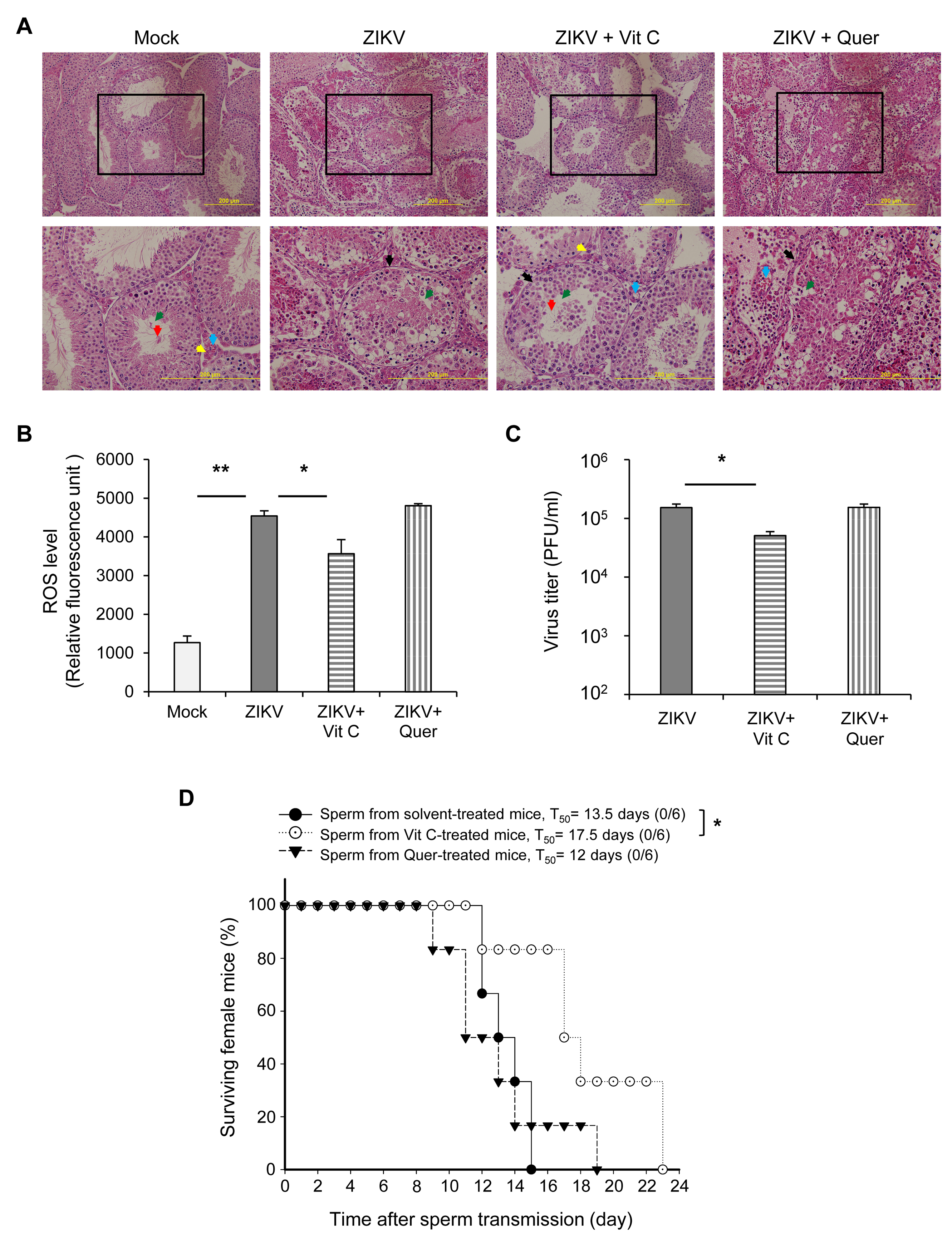

Supplement: S6 Fig — (A) Histological analysis of testis sections stained with haematoxylin and eosin. Arrows indicate lumen (green), sperm (red), blood capillary (blue), interstitial cell (yellow), and degeneration of SNT (black). Scale bar: 200 μm. (B) Intracellular ROS assay. ROS levels in sperm were measured by use of the OxiSelect intracellular ROS indicator. Relative fluorescence intensity was determined by use of a fluorescence plate reader. (C) Plaque-forming assay of viral load in sperm. (D) Survival of female mice receiving semen transfer. Testes and sperm were collected on day 9 after infection. Data are mean ± SD (n = 6 mice/group). *P<0.05 and **P<0.01 by Kruskal-Wallis, Bonferroni post-hoc test. The median survival time (T50) is presented. Survival curves of female mice were compared by Log-rank test (*P = 0.01). Abbreviation: Vit C, vitamin C; Quer, quercetin. (TIF) [file ppat.1006854.s006.tif]
